# IHGA: An interactive web server for large-scale and comprehensive discovery of genes of interest in hepatocellular carcinoma

**DOI:** 10.1016/j.csbj.2023.08.003

**Published:** 2023-08-06

**Authors:** Qiangnu Zhang, Weibin Hu, Lingfeng Xiong, Jin Wen, Teng Wei, Lesen Yan, Quan Liu, Siqi Zhu, Yu Bai, Yuandi Zeng, Zexin Yin, Jilin Yang, Wenjian Zhang, Meilong Wu, Yusen Zhang, Gongze Peng, Shiyun Bao, Liping Liu

**Affiliations:** a Division of Hepatobiliary and Pancreas Surgery, Department of General Surgery, Shenzhen People’s Hospital (The Second Clinical Medical College, Jinan University, The First Affiliated Hospital, Southern University of Science and Technology), 518020 Shenzhen, China; b Integrated Chinese and Western Medicine Postdoctoral Research Station, Jinan University, 510632 Guangzhou, China; c Key Laboratory of Brain, Cognition and Education Sciences, Ministry of Education, Institute for Brain Research and Rehabilitation, South China Normal University, 510631 Guangzhou, China; d Department of Hepatobiliary Surgery, The First Affiliated Hospital of Guangdong Pharmaceutical University, 510632 Guangzhou, China; e Department of Neurology, Shenzhen People’s Hospital (The Second Clinical Medical College, Jinan University, the First Affiliated Hospital, Southern University of Science and Technology), 518020 Shenzhen, China; f Cytotherapy Laboratory, Shenzhen People’s Hospital (The Second Clinical Medical College, Jinan University, the First Affiliated Hospital, Southern University of Science and Technology), 518020 Shenzhen, China; g Laboratory Medicine Center, Huazhong University of Science and Technology Union Shenzhen Hospital (Nanshan Hospital), 518000 Shenzhen, China

**Keywords:** Hepatocellular carcinoma, Data mining, Gene expression profile, Prognosis, Web tools

## Abstract

Mining gene expression data is valuable for discovering novel biomarkers and therapeutic targets in hepatocellular carcinoma (HCC). Although emerging data mining tools are available for pan-cancer–related gene data analysis, few tools are dedicated to HCC. Moreover, tools specifically designed for HCC have restrictions such as small data scale and limited functionality. Therefore, we developed IHGA, a new interactive web server for discovering genes of interest in HCC on a large-scale and comprehensive basis. Integrative HCC Gene Analysis (IHGA) contains over 100 independent HCC patient-derived datasets (with over 10,000 tissue samples) and more than 90 cell models. IHGA allows users to conduct a series of large-scale and comprehensive analyses and data visualizations based on gene mRNA levels, including expression comparison, correlation analysis, clinical characteristics analysis, survival analysis, immune system interaction analysis, and drug sensitivity analysis. This method notably enhanced the richness of clinical data in IHGA. Additionally, IHGA integrates artificial intelligence (AI)–assisted gene screening based on natural language models. IHGA is free, user-friendly, and can effectively reduce time spent during data collection, organization, and analysis. In conclusion, IHGA is competitive in terms of data scale, data diversity, and functionality. It effectively alleviates the obstacles caused by HCC heterogeneity to data mining work and helps advance research on the molecular mechanisms of HCC.

## Highlights

• IHGA currently includes the largest number of HCC datasets and samples among similar online tools.
• IHGA enhances clinical feature analysis, improving the translational value of screening results.
• IHGA facilitates HCC biomarker screening and identifies potential therapeutic targets, aiding mechanism research with supporting evidence.
• IHGA's comprehensive analysis offsets HCC data mining drawbacks caused by heterogeneity, enabling large-scale insights.

## 1 Introduction

Hepatocellular carcinoma (HCC) is the second leading cause of cancer-related death worldwide and accounts for more than 90% of primary liver cancers [1], [2], [3]. Approximately 800,000 new cases and 700,000 deaths are reported each year as a result of HCC, which imposes a substantial burden on global health [4]. HCC patients experience unsatisfactory long-term survival owing to the rapid growth, high frequency of metastasis, treatment resistance, and recurrence [5], [6], [7]. Hence, it is necessary to fully understand the detailed molecular mechanisms of HCC progression and discover novel therapeutic strategies or strengthen current treatments. Over the past decade, a vast amount of HCC-related transcriptomic data has been accumulated, which has facilitated the investigation of molecular mechanisms underlying HCC. This unique opportunity has given researchers valuable resources for identifying potential therapeutic targets and biomarkers for HCC [8], [9]. For instance, Wu et al. performed a comprehensive bioinformatics analysis to establish an RNA-binding motif protein family-based prognostic signature for HCC using patient samples data from The Cancer Genome Atlas (TCGA) and International Cancer Genome Consortium (ICGC) [10]. Cai et al. analyzed lactylation-related genes in an integrative way and established a novel prognostic signature for using public HCC datasets [11]. Based on HCC data from databases such as TCGA and Gene Expression Omnibus (GEO), ALDH2 has been identified as a prognostic biomarker that is related to immune infiltrates in HCC [12]. However, effectively and efficiently utilizing these data, especially for large-scale datasets and integrative data mining, presents a significant challenge for most HCC researchers.

Over the past five years, a plethora of online tools for mining cancer data have been developed, including well-known products such as Xena [13], cBioPortal [14], and GEPIA [15]. These tools promote reliable and accurate discovery of tumor biomarkers and expedite their clinical translation. However, most of these tools have been designed for pan-cancer analysis, and tools for HCC-specific data are lacking. Furthermore, the datasets incorporated by these tools are largely derived from TCGA program; thus, the currently available online tools for HCC data analysis primarily rely on the TCGA liver HCC cohort (TCGA-LIHC). Notably, HCC is a highly heterogeneous tumor, and therefore, the single TCGA-LIHC cohort is insufficient to meet the requirements of HCC researchers despite its high quality and significant reference value. Some teams have dedicated themselves to developing gene data analysis tools specializing in HCC, such as the Hepatocellular Carcinoma Expression Atlas Database (HCCDB), which was released in 2018 [16]. However, this tool only includes 15 cohorts and has limited functionality. CancerLivER is another gene expression resource and biomarker database for liver cancer [17]. Although this dataset is larger than HCCDB, the interface has limited functionality and is less intuitive. CancerLivER only allows for comparisons of gene expression levels and lacks commonly used analyses related to correlation and prognosis.

Building on the strengths of the aforementioned tools and databases, we have developed Integrative HCC Gene Analysis (IHGA), an intuitive and interactive web server for analyzing gene mRNA expression in HCC. Using IHGA, we attempted to overcome the limitations of the existing tools by providing a more user-friendly interface and incorporating additional analyses related to correlation and prognosis. IHGA allows more than 100 HCC patient-derived datasets to be integrated, including over 10,000 tissue samples, which increases the representation of HCC heterogeneity. To our knowledge, this represents the largest patient sample size among the current HCC-related analysis tools or databases. Based on this large-scale dataset, users can perform comprehensive analysis of mRNA expression levels and correlation. Specifically, we have enhanced the analysis functions related to clinical features and prognosis. Compared with the existing databases and tools, IHGA enables more comprehensive analyses of clinical features and incorporates more analysis modules related to prognosis. In addition to the commonly studied immune microenvironment correlation analysis, we have also included analysis of drug sensitivity based on HCC, which is rarely seen in the existing tools. IHGA also offers a variety of visualization options and easily editable result outputs to facilitate data interpretation. IHGA is available at https://www.hccdatasph.cn/app/ihga.

## 2 Material and methods

### 2.1 Implementation

**Fig. 1:**
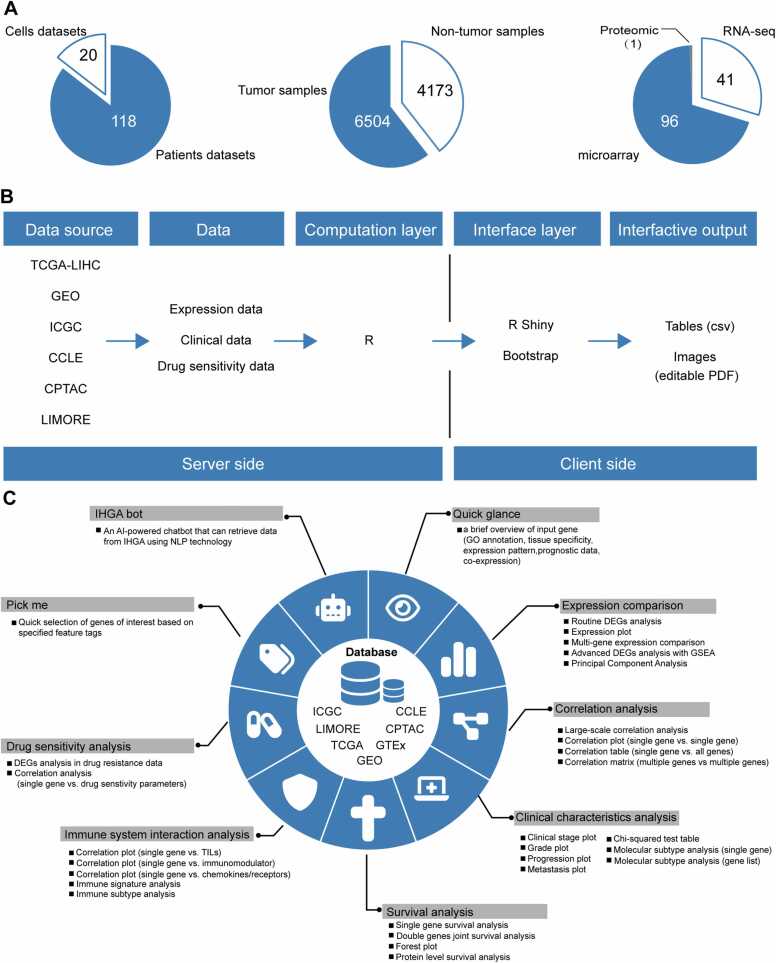
IHGA overview. (A) The available data size and distribution composition collected by IHGA for analysis; (B) Schema describing data processing and data display for the IHGA ONLINE tool. (C) IHGA function map.

Except for internal team data related to the resistance of HCC cells to lenvatinib, all other data sources of IHGA are publicly available. RNA sequencing and microarray data obtained from public datasets were subjected to quality control, preprocessing, and standardization using conventional methods. The data size and distribution of IHGA is shown in Fig. 1A. IHGA is a completely free online app that can be accessed without registration. On the server side, data analysis and computation are based on R scripts (Version 4.2.2) and corresponding R packages. All data analysis is based on standard analysis pipelines. The client-side is built using R shiny (Version1.7.4) and Bootstrap (Version 5.2.3). The output results include tables and images, with tables in standard CSV format and images in editable PDF format. Users can edit the image style (including the color and font size) as desired. The overall architecture of the app is shown in Fig. 1B. The artificial intelligence (AI)-assisted data analysis function (IHGA bot) is provided by My ASKAI (https://myaskai.com/). To deploy IHGA and enable parallel user access, ShinyProxy (Version 3.0.1) and Docker (Version23.03) are used.

The R packages used include shinydashboard, shinycssloaders, shinydashboardPlus, shinyWidgets, and htmltools to generate the exchange interface; wordcloud2 to generate the word cloud on the homepage; visNetwork to generate the network graph; DT to generate tables; ggplot2, ggpubr, pheatmap, ggrepel, and forestplot for data visualization; data.table for data reading; dplyr for data preprocessing; survival for survival analysis; survminer for generating analysis; limma for differential analysis; clusterProfiler for GSEA analysis; and org.Hs.eg.db for gene annotation. All R packages were downloaded from Comprehensive R Archive Network (CRAN; https://cran.r-project.org/) and Bioconductor (https://bioconductor.org/).

The main functions of IHGA can be divided into the following sections: quick glance, expression comparison, correlation analysis, clinical characteristics analysis, survival analysis, immune system interaction analysis, drug sensitivity analysis, pick me, and IHGA bot. Please refer to Fig. 1C for detailed information on the functions of each section.

## 3 Results

Solute carrier family 25 member 15 (SLC25A15) is involved in the urea cycle by encoding the protein that transports ornithine across the inner mitochondrial membrane from the cytosol to the mitochondrial matrix [18], [19]. It is a tumor suppressor gene, and our team has discovered that it has anticancer effects in HCC. In this section, the analysis and exploration process of SLC25A15 is used as a case study to demonstrate the primary functionalities of IHGA. Although these examples demonstrate only some of the primary functionalities of IHGA, here we show how to use IHGA to screen and validate HCC-related genes of interest, and how to use IHGA to gain insights into the mechanisms underlying HCC.

### 3.1 Home page and quick glance

**Fig. 2:**
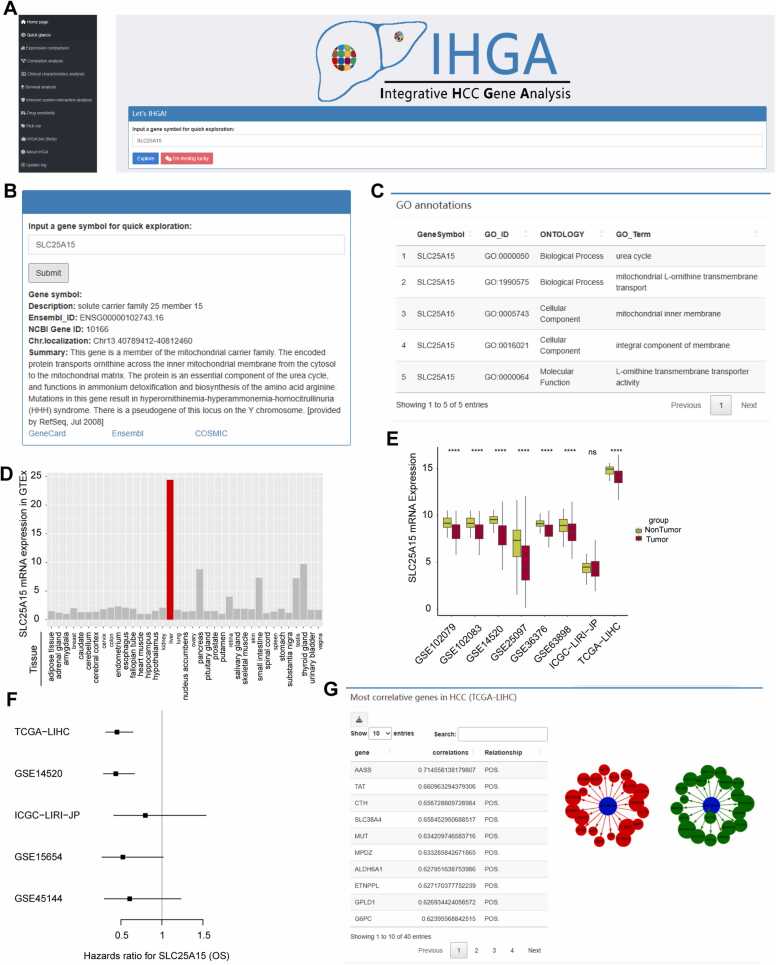
A screenshot of the IHGA home page and an example of the quick glance function. (A) A screenshot of the home page; (B) In the quick glance function, basic descriptive information about the SLC25A15 gene is provided. Users can access more information on SLC25A15 directly in other databases through the external links provided below; (C) The results of the gene oncology analysis of the SLC25A15 gene are displayed, and users can obtain information on the molecular function, cellular localization, and biological function of SLC25A15; (D) The expression of SLC25A15 in different tissues is displayed in the GTEx dataset; (E) The expression levels of SLC25A15 mRNA in tumor and nontumor tissues are compared in a selection of eight HCC cohorts; (F) The forest plot displays the relationship between SLC25A15 mRNA levels and overall patient survival. OS: overall survival; (G) The top 20 genes most correlated with SLC25A15 in TCGA-LIHC are listed and visualized using a network graph.

On the IHGA home page, users can select various functionalities through the sidebar or enter the genes they wish to explore (Fig. 2A), which will then direct them to the “quick glance” function. In quick glance, users can receive a rapid report on various features of the input gene, allowing them to form a preliminary impression of the gene. Taking SLC25A15 as an example, the quick glance feature provides basic gene information (Fig. 2B) and gene ontology (GO) annotations of SLC25A15, which assists users in understanding the basic functions and subcellular localization of SLC25A15 (Fig. 2C). Users can also access more information on SLC25A15 through external links to recognized databases. The mRNA expression plot of SLC25A15 in GTEx displays high specificity in liver tissue (Fig. 2D), while in selected datasets (several frequently reported HCC datasets), the expression level of SLC25A15 mRNA is significantly reduced in HCC tissues (Fig. 2E). The forest plot based on the Cox proportional hazards model in Fig. 1F suggests that low levels of SLC25A15 are a risk factor for overall survival. Finally, quick glance lists and visualizes the genes most correlated with SLC25A15 mRNA levels (Fig. 2G). These results indicate that SLC25A15 likely plays a tumor suppressor role in HCC.

### 3.2 Expression comparison analysis

**Fig. 3:**
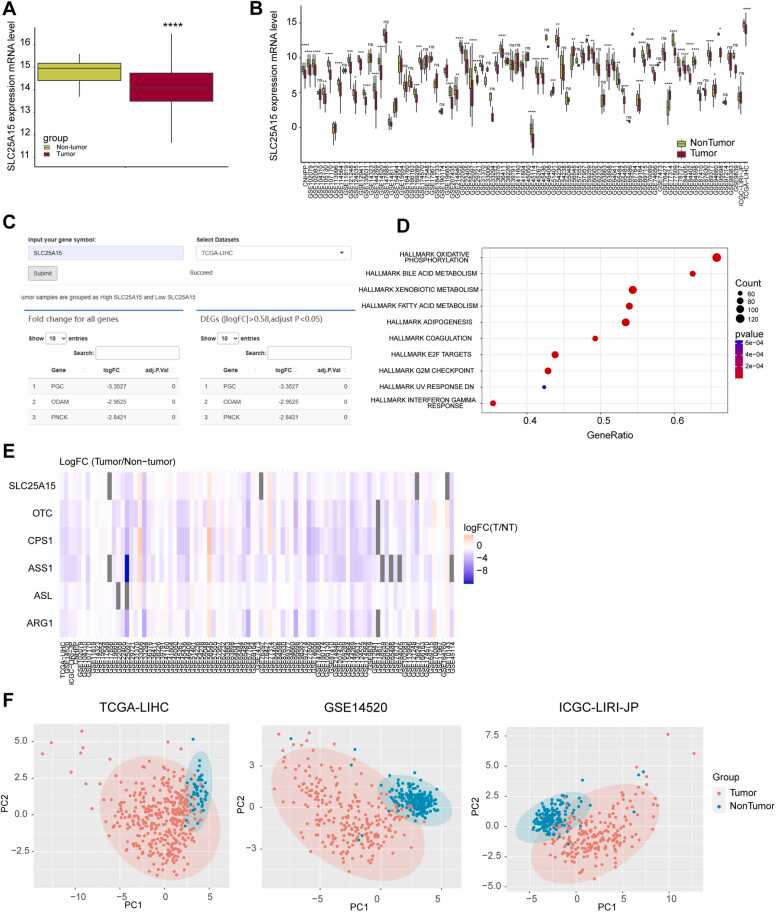
Examples of expression comparison analysis. (A) The mRNA expression of SLC25A15 in tumor and nontumor tissue samples is displayed in a single selected HCC dataset (TCGA-LIHC); (B) The mRNA expression of SLC25A15 in tumor and nontumor tissue samples is comprehensively compared by including all available HCC datasets for analysis simultaneously; (C) The mRNA expression of SLC25A15 in tumor and nontumor tissue samples is comprehensively compared by including all available datasets for analysis simultaneously; (D) Gene enrichment analysis based on expression fold change is performed for the genes in tumor tissue of patients with high SLC25A15 and those with low SLC25A15, and the top 10 enrichment results are visualized as a bubble plot; (E) The expression trends of SLC25A15 and other key genes involved in the urea cycle regulation are integratively analyzed in all available HCC datasets. A heat map is displayed to show the expression differences of these genes between tumor and nontumor tissues; (F) Dimensionality reduction analysis is performed using principal component analysis (PCA), which includes a series of selected SLC family genes, including SLC25A15. *P < 0.05; * *P < 0.01; * **P < 0.001; * ** *P < 0.0001; ns: no statistical significance.

In the expression comparison analysis feature, users can perform a more in-depth analysis of the expression levels of SLC25A15. In the ‘Expression plot’ tab, users can plot the expression of SLC25A15 in a single dataset (Fig. 3A) or select all 91 datasets to eliminate the bias caused by HCC heterogeneity and obtain more accurate results (Fig. 3B). To investigate the downstream regulatory mechanisms of SLC25A15 in HCC, users can access the ‘Grouped by a gene’ tab, which provides an advanced differential gene analysis. For example, if the TCGA-LIHC dataset is selected, HCC patients are divided into low and high SLC25A15 expression groups based on the median SLC25A15 mRNA expression, and the differentially expressed genes between the two groups are extracted (Fig. 3C). Furthermore, gene set enrichment analysis (GSEA) and visualization are performed based on this grouping, and pathways (hallmarker gene sets selected) that show differences between the high and low SLC25A15 expression groups are displayed (Fig. 3D). This provides a reference for selecting the downstream regulatory pathways of SLC25A15. The expression comparison analysis feature also provides a multi-gene expression analysis. SLC25A15 is a gene that controls the urea cycle, and we selected several other genes related to the urea cycle to demonstrate IHGA's functionality for comparing the expression levels of multiple genes, as shown in Fig. 3E. SLC25A15 and other urea cycle–related genes show a low-expression trend in most HCC datasets, which is consistent with literature reports that the urea cycle is disrupted in HCC [20]. This feature is useful for understanding a group of functionally similar or related genes. IHGA also provides a dimensionality reduction analysis based on mRNA levels of a given gene list. This feature can confirm whether a gene set can distinguish between tumor and nontumor tissues, thereby determining whether this set of genes can be used as effective HCC biomarkers. We input other members of the SLC family that are related to HCC, along with SLC25A15, which were identified in previous studies, into the dimensionality reduction analysis. As shown in Fig. 3F, this gene set can be used to distinguish between tumor and nontumor tissues in selected datasets (TCGA-LIHC, GSE14520, and ICGA-LIRI-JP). This suggests that SLC members, including SLC25A15, have the potential to serve as HCC biomarkers.

### 3.3 Correlation analysis

**Fig. 4:**
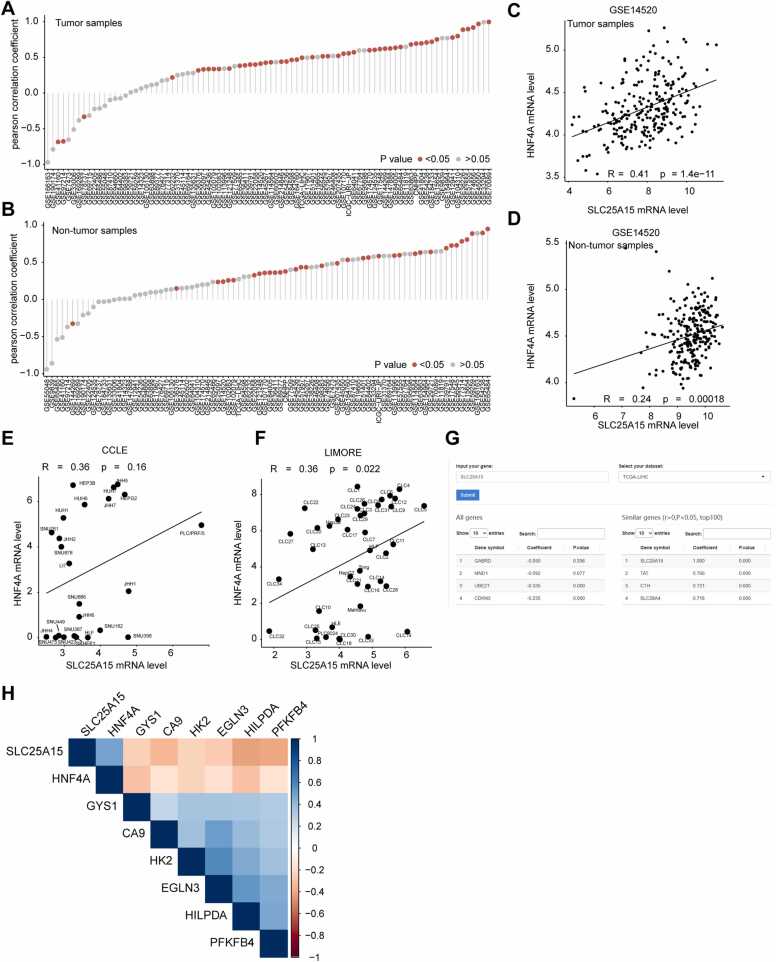
Examples for correlation analysis. (A–B) The correlation between SLC25A15 and HNF4A mRNA levels is calculated in both tumor and nontumor tissues by comprehensively including all available HCC datasets; (C–D) The correlation between SLC25A15 and HNF4A mRNA levels is calculated and visualized as a scatter plot in both tumor and nontumor tissues in the user-selected dataset (GSE14520); (E–F) RNA-sequencing data from cell models collected from CCLE and LIMORE datasets are used to obtain mRNA expression data for SLC25A15 and HNF4A genes in the cell models. The correlation between SLC25A15 and HNF4A mRNA levels is calculated and visualized as a scatter plot; (G) All genes that may have expression correlation with SLC25A15 are screened and listed in a table in the specified dataset (TCGA-LIHC); (H) The correlation matrix of the expression of SLC25A15, HNF4A, and several common hypoxia marker genes is calculated and displayed in a heat map.

Correlation analysis is a routine process in gene data mining that can help identify the upstream regulatory or downstream target genes of a gene. We found that HNF4A, a member of the nuclear receptor family of transcription factors [21], [22], can promote the transcription of SLC25A15 mRNA in the cell model, and IHGA can be used to search for tissue-level evidence. IHGA can be used to perform correlation analysis for two specified genes across multiple datasets, as shown in Fig. 4A–B. Users can choose from three correlation calculation methods, including Pearson, Spearman, and Kendall. In most HCC datasets, both in tumor and nontumor tissues, SLC25A15 and HNF4A are significantly positively correlated. Users can select specific datasets (e.g., GSE14520) and generate scatter plots to reflect the correlation between SLC215A15 and HNF4A in tumor or nontumor samples (Fig. 4C–D). External cell model data collected by IHGA can also be used to confirm the correlation between SLC25A15 and HNF4A (Fig. 4E–F), providing additional evidence for the user's academic hypothesis. If users do not yet have candidate genes, they can use the 'Gene vs all' tab to obtain all candidate genes related to their specified gene in the selected datasets (Fig. 4G). In the 'correlation matrix' tab, users can perform correlation analysis and visualize the correlation matrix among two or more genes. For example, we found that hypoxia can inhibit SLC25A15 mRNA expression by reducing HNF4A levels in the cell model. To find more evidence, we used IHGA to obtain the correlation matrix among SLC25A15, HNF4A, and common hypoxia marker genes [23], [24], as shown in Fig. 4H. The correlation matrix intuitively displays that SLC25A15, HNF4A, and hypoxia marker genes are inversely correlated, which increases the credibility of our cell model data.

### 3.4 Clinical characteristic analysis

**Fig. 5:**
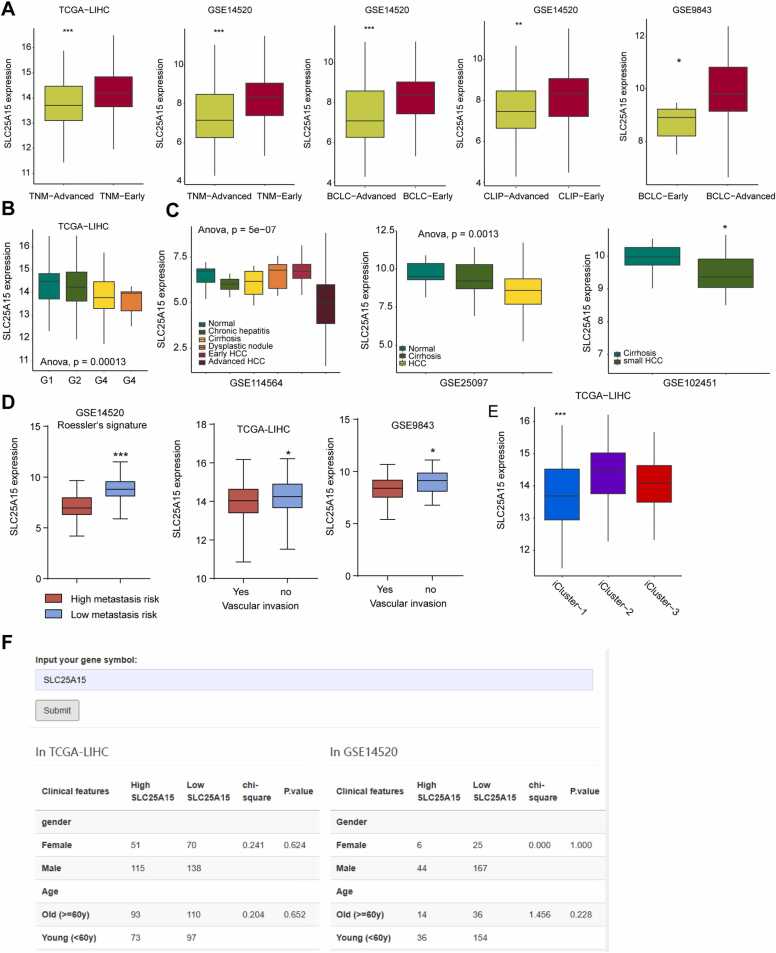
Examples of clinical characteristics analysis. (A) The levels of SLC25A15 mRNA were compared between tumor tissues from early-stage and those from late-stage patients. Users can choose different staging systems from the available datasets; (B) The levels of SLC25A15 mRNA were compared between cancer tissues with different degrees of differentiation. In this case, cancer tissue specimens from patients with different grades were selected from TGCA-LIHC dataset; (C) The changes in mRNA expression levels of SLC25A15 during different progression of HCC formation are displayed; (D) The levels of SLC25A15 mRNA were compared among tumor tissues from patients with different invasive characteristics; (E) The levels of SLC25A15 mRNA were compared among tumor tissues from patients with different molecular subtypes; (F) Patients were divided into two groups, namely, high SLC25A15 group and low SLC25A15 group, based on the median level of SLC25A15 mRNA in tumor tissues. Chi-square test was used to analyze the differences in common pathological features between the two groups of patients, and the results are listed in a table. * *P* < 0.05; * *P* < 0.01; * **P < 0.001.

The relationship between genes, clinical pathological features, and prognosis is the most important aspect that researchers focus on in the process of screening for HCC biomarkers or therapeutic targets [25], [26], [27], [28]. Compared with similar tools (e.g., GEPIA and HCCDB), IHGA has strengthened the analysis function in this area and added available clinical data. As shown in Fig. 5A, IHGA can display the differential expression of SLC25A15 mRNA in tumor tissues of early- and late-stage patients in different clinical staging systems across multiple datasets. Grade data indicate that SLC25A15 is significantly downregulated in poorly differentiated tumor tissues in TCGA-LIHC (Fig. 5B). The development of HCC is a gradual process, and the hepatitis–cirrhosis–HCC pathway is a classic progression pathway for HCC [29], [30], [31]. In the ‘clinical progression analysis’ module, the stepwise changes in gene expression among the processes of HCC formation are displayed. This can help to deepen our understanding of the molecular mechanisms underlying the development of HCC, and thereby identify potential strategies for blocking its progression. We found that the decrease of SLC215A15 is associated with the genesis and development of HCC (Fig. 5C). Based on the 'metastasis' module data, low SLC25A15 is also associated with the invasive features of HCC (Fig. 5D). IHGA has also added expression analysis of genes in HCC molecular subtypes. As shown in Fig. 5E, SLC25A15 is downregulated in patients with rapid-proliferation icluster1-type HCC [32]. IHGA can also be used to group patients and perform chi-square tests on pathological features according to the median level of SLC25A15, which is a common user requirement in HCC-related research (Fig. 5F).

### 3.5 Survival analysis

**Fig. 6:**
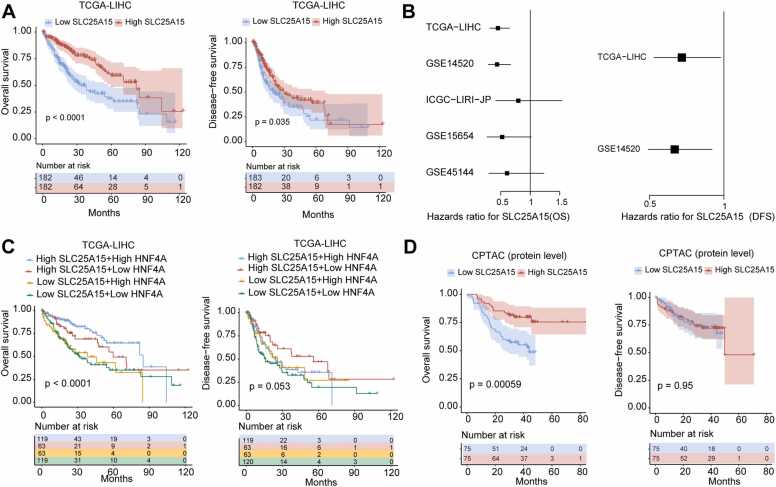
Examples of survival analysis. (A) Patients were divided into two groups, namely, high SLC25A15 group and low SLC25A15 group, based on the median level of SLC25A15 mRNA in tumor tissues (users can also choose other values as the cutoff). Kaplan–Meier survival curves were used to analyze the relationship between SLC25A15 and overall survival or disease-free survival rates of patients in the selected dataset (TCGA-LIHC). (B) Univariate Cox proportional hazards model was used to analyze the relationship between SLC25A15 mRNA levels and overall survival or disease-free survival rates of patients in the selected dataset. The results are visualized using a forest plot; (C) The median values of SLC25A15 and HNF4A mRNA were combined to group patients in the selected dataset. Kaplan–Meier survival curves were used to analyze the relationship between SLC25A15/HNF4A and overall survival or disease-free survival rates of patients in the selected dataset (TCGA-LIHC); (D) Patients were divided into two groups, namely, high SLC25A15 group and low SLC25A15 group, based on the median level of SLC25A15 protein in tumor tissues. Kaplan–Meier survival curves were used to analyze the relationship between SLC25A15 and overall survival or disease-free survival rates of patients in the CPTAC dataset.

In the survival analysis section, users can utilize the expression level of SLC25A15 to group patients in multiple datasets and explore the relationship between SLC25A15 and overall survival or disease-free survival in seven datasets. IHGA provides Kaplan–Meier survival curves (Fig. 6A, TCGA-LIHC selected as an example, cutoff = median value of SLC25A15) and forest plots for visualization (Fig. 6B). We also added a survival analysis tool based on dual-gene grouping to IHGA. For example, we used SLC25A15 and its upstream regulatory gene HNF4A to group patients, and the results showed that patients with low expression of both SLC25A15 and HNF4A had the worst overall and disease-free survival (Fig. 6C), suggesting the important role of the HNF4A/SLC25A15 pathway in HCC. We also added collected data from CPTAC, which provides protein-level survival analysis, to IHGA (Fig. 6D).

### 3.6 Drug sensitivity analysis

**Fig. 7:**
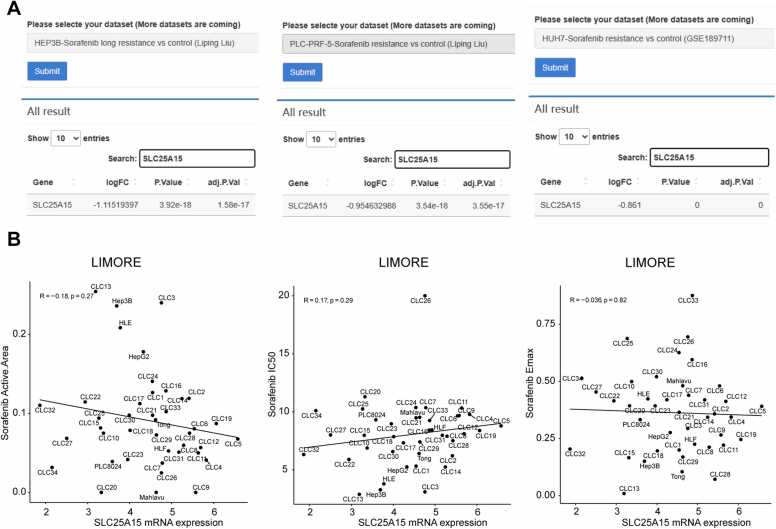
Examples of drug sensitivity analysis. (A) mRNA expression data of various drug-resistant cell models in HCC were collected in IHGA, and differentially expressed genes were extracted. In this case, the mRNA expression levels of SLC25A15 are shown in three HCC cell lines resistant to sorafenib; (B) The correlation between SLC25A15 mRNA levels and three sensitivity parameters to sorafenib was analyzed, and the results are shown as a scatter plot.

IHGA offers drug-related gene expression data analysis, including differential gene expression analysis with regard to common HCC drug resistance and correlation analysis between gene expression levels and drug sensitivity indicators. These tools help to deepen the understanding of specific genes and improve related drug therapies. For example, we found that SLC25A15 is significantly downregulated in multiple sorafenib-resistant HCC cell models (Fig. 7A), suggesting that low SLC25A15 may induce sorafenib resistance in HCC. Unfortunately, in the sensitivity parameter correlation analysis, the data from LIMORE did not suggest a correlation between SLC2515A and sorafenib sensitivity parameters (Fig. 7B). Such inconsistencies between the analyses are normal, and IHGA is based on objective data and provides analysis functions accordingly. However, IHGA cannot explain such contradictions between different analyses, so users should rely on their knowledge or actual situation.

### 3.7 Additional characteristic functions to quickly find genes of interest

**Fig. 8:**
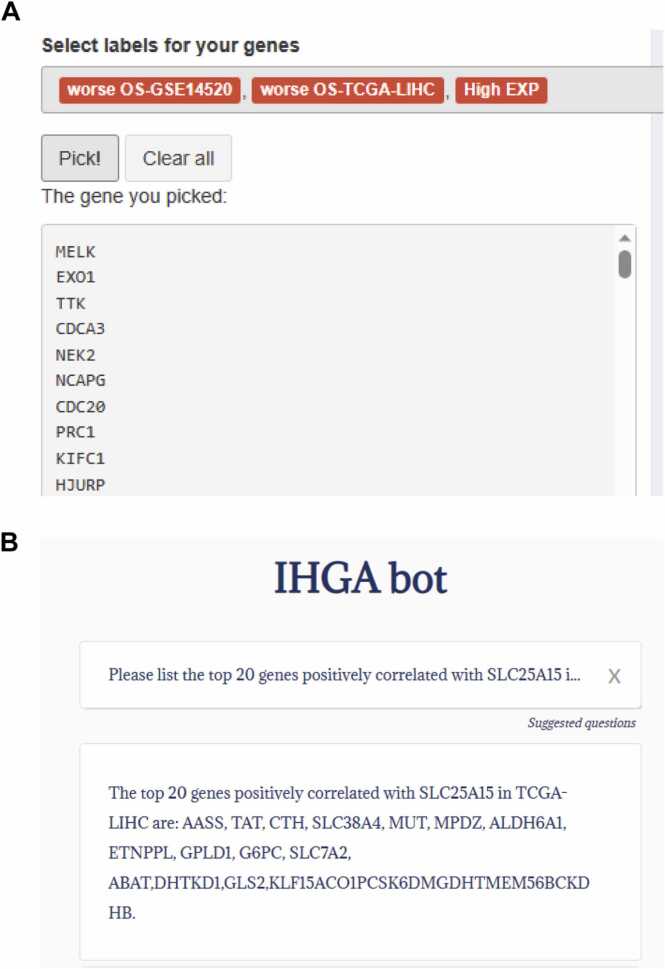
Examples of pick me and IHGA bot. (A) The user screens genes based on three selected feature labels, and the genes that meet the criteria are listed; (B) The user may ask IHGA bot in natural language for a list of genes that correlate with SLC25A15 in the TCGA-LIHC dataset, and receives feedback.

Pick me: This function is used to quickly filter genes that meet certain criteria based on predefined feature labels. The feature labels currently include gene expression characteristics, their relationship with prognosis, and liver specificity; more labels are being added continuously to enrich the feature set. As shown in Fig. 8A, by selecting three feature labels, all genes that are highly expressed in multiple HCC datasets (high EXP) and associated with unfavorable overall survival in both GSE14520 (worse OS-GSE14520) and TCGA-LIHC (worse OS-TCGA-LIHC) datasets are filtered out. This suggests that this feature can help quickly identify both oncogenes and tumor suppressor genes in HCC.

IHGA bot: This is AI-assisted querying based on natural language model service. Users can explore the data included in IHGA through a chat interface, as shown in Fig. 8B. This feature is still in the testing phase, but it represents an advance in the development of online data mining tools.

## 4 Discussion

Using publicly available data to screen and identify biomarkers of HCC is a commonly used technique. For example, Yan’s team used a comprehensive bioinformatics analysis to analyze the correlation between cancer-associated RNA methylation regulators and HCC malignant features in public HCC datasets [33]. In our previous study, we conducted an integrated analysis of multiple HCC datasets from various databases. Through this analysis, we identified a series of genes belonging to the RNA-binding protein family that could serve as prognostic biomarkers of HCC [34]. In the study conducted by Chen et al., a comprehensive analysis was performed using databases; it was found that KLF2 could serve as a prognostic biomarker associated with fibrosis and immune infiltration in advanced HCC [35]. We hope that the emergence of IHGA will significantly accelerate the progress of such research. IHGA integrates a dataset derived from 118 HCC patients, comprising 4136 nontumor tissues and 6344 tumor tissues. Additionally, IHGA encompasses gene expression data from 96 cell models in addition to 92 drug-related gene expression data. To our knowledge, IHGA represents the most extensive online gene analysis tool for HCC in terms of the scale of the datasets it incorporates. In addition to the larger scale of expression data, compared with the existing tools, IHGA also represents an expansion in terms of clinical data. Most online analysis tools related to HCC genes only contain clinical data from TCGA-LIHC, GSE14520, and ICGC-LIRI-JP, and only survival data are available [16]. However, we not only included clinical data from a greater number of datasets, but also added analysis functions for clinical staging and invasive features. The development of liver cancer is a gradual process, and our 'progression' function can investigate gene expression changes during liver cancer formation using multiple HCC datasets. For example, we can study the expression changes of a gene in the progression process from normal–chronic hepatitis–cirrhosis–dysplastic nodule–early HCC to advanced HCC. When performing gene analysis using the same dataset, our tool is able to provide more information than the existing tools. Users can analyze gene differences and correlations from multiple perspectives, and they have access to more adjustable parameters to meet their specific requirements. However, these additions do not increase the complexity of the tool. In IHGA, most analyses can be performed through the integration of multiple independent datasets, which, to a certain degree, mitigates potential data inaccuracies arising from HCC heterogeneity [36].

IHGA currently has limitations, but it is continually evolving. First, the data source will be continually expanded, including both the data scale and data type. We will continuously update the data source, introducing the latest data. In this version, we only included data on coding genes, but in the next stage, we plan to include noncoding RNA–related data, such as lncRNA, microRNA, and circRNA. Furthermore, we aim to incorporate multiple omics data, such as genomics, epigenetics, and metabolomics, to further emphasize the concept of integrated analysis. The inclusion of single-cell sequencing data is also under consideration. Second, we aim to enhance the visualization function, so although users can currently download editable PDF files for customization, we will gradually add online functionality for modifying image styles, further saving user time. Certain types of plots will also be gradually added. Finally, we wish to explore strengthening the AI functionality in IHGA. Our vision is that in future versions, users can bid farewell to mouse clicks and engage in in-depth analysis and faster visualization through chat-based interactions. AI is expected to play a significant role in improving the utility of IHGA in the future. For instance, AI can help identify patterns and relationships in complex datasets, perform predictive modeling, and aid in data interpretation and visualization. This may help researchers better understand data and make more informed decisions.

In summary, IHGA represents a convenient and efficient HCC gene expression analysis tool that seamlessly integrates large-scale datasets to furnish researchers with more comprehensive and precise HCC gene expression data. This, in turn, could expedite the discovery of novel HCC biomarkers and molecular therapeutic targets. A prominent advantage of IHGA lies in its ability to economize a significant quantum of time and effort, particularly for researchers who lack programming acumen. It is anticipated that IHGA will attain a pervasive presence in the routine data analysis workflow of HCC researchers.

## Ethics approval and consent to participate

Not applicable.

## Funding

This study was supported by the 10.13039/501100002858China Postdoctoral Science Foundation (2021M701426), GuangDong Basic and Applied Basic Research Foundation (2022A1515110283), Shenzhen Science and Technology Innovation Commission Foundation (JCYJ20190806160412946 and JCYJ20210324113008022), Guangdong Basic and Applied Basic Research Foundation (2021A1515220059, 2019A1515110149), the 10.13039/501100001809National Natural Science Foundation of China (82002956, 8140204), Shenzhen Key Medical Discipline Construction Fund (No. SZXK015), Guangdong Provincial and National Key Clinical Specialty Construction Project and National Key Clinical Specialty Construction Project, and Training Program for cultivating clinical physician-scientists of Shenzhen People's Hospital (SYWGSJCYJ202203).

## CRediT authorship contribution statement

ZQN built the server base system; ZQN, HWB, and XLF designed the user interface; JW, XLF, and HWB collected and cleaned the data; WT, YLS, LQ, ZSQ, and BY proposed functional requirements and provided solutions; ZYL, YZX, YJL, PGZ, and ZWJ tested and debugged the app; WML, PGZ, and ZYS assisted with the deployment of the app's server; BSY and LPL supervised the project.

## Declaration of Competing Interest

No potential conflict of interest was reported by the authors.
